# Mathematical model for volcanic harmonic tremors

**DOI:** 10.1038/s41598-019-50675-2

**Published:** 2019-10-08

**Authors:** Giordano Montegrossi, Angiolo Farina, Lorenzo Fusi, Antonietta De Biase

**Affiliations:** 1 IGG Istituto Geoscienze e Georisorse, CNR, Via la Pira 4, Firenze, 50121 Italy; 2 Consorzio Interuniversitario Nazionale per la Scienza e Tecnologia dei Materiali, INSTM, Via G. Giusti 9, Firenze, 50134 Italy; 3 Dipartimento di Matematica e Informatica “U. Dini”, Viale Morgagni 67/a, Firenze, 50134 Italy

**Keywords:** Seismology, Volcanology, Geophysics

## Abstract

Harmonic tremors consist in the release of infrasonic energy associated with volcanic activity. The typical frequency range of harmonic tremors is 0.1–12 Hz. We suppose that the harmonic tremors are due to the formation of bubbles entrapped in cavities that oscillate converting thermal energy into mechanic energy. Reproducing the natural phenomenon through an experimental apparatus, we propose here a mathematical model to describe the oscillatory mechanism and to detect the frequency as a function of the main physical parameters. We show that the frequency obtained through the model is in agreement with the one obtained through experimental measurements and with the data available from the literature, proving the consistency of the proposed model.

## Introduction

Harmonic tremors with frequency 0.1–12 Hz often occur in volcanos related to eruptive phenomena, but they can also be present in volcanoes without any unusual activity^1^. Infrasounds are commonly reported as time series or spectrograms, to show the evolving frequency content in a signal^2,3^. Some of these tremors are attributed to being the result of more and more rapidly occurring pulses, while others are the result of an explosion. Finally, some harmonic tremors just emerge and disappear apparently not having a known cause.

The measurement of acoustic (infrasound) signals is important to localize the source of tremors and to gather a deeper insight on the phenomena responsible of their emergence^4^. The source is localized using a grid searching analysis as reported in^4^. To understand the dynamics of volcanic tremors, the fumarole thermal trends can also be studied. Thermal measurements are generally done by using infrared thermometers, and by investigating the non-linear behaviour in the temperatures we can discover the link between multiple fumaroles and the effect of the fluid dynamics at depth.

Recently Busse *et al*.^5^ have proposed that harmonic tremors are linked to thermoacoustic instabilities, i.e. the oscillations of large gas bubbles entrapped in cracks. This association is quite straightforward because volcanoes are surrounded by hydrothermal reservoirs, namely rock cracks in which vapor or gas bubble are heated by the magma. We however have to remark that the causes of the harmonic tremors are still uncertain and that a better understanding of possible sources would be a further step in expanding our knowledge^6–10^.

There are many non-linear behaviors possible in a volcanic system related to both fluid flow and chemical system, as commented e.g. in^11^. During the fast uprising of the gas, steam, liquid droplets and solid particles the gas temperature may span in the interval 80–1100 °C and turbulent and laminar motion alternate, generating oscillations related to fluid dynamic features and posing a challenge to the fluid sampling^11–15^.

A recent investigation^16^ carried out at Pisciarelli fumarole, Campi Flegrei volcano (Italy), reports the data from a seismic station located at 8 m distance from the fumarole. Polarization analysis results indicate that the signal is dominated by the vertical component, and originates from the vent. Its prominent spectral peak at ~10 Hz frequency is stable over time. A characteristic RSAM (Real-time Seismic-Amplitude Measurement) value is computed daily by assuming as representative the minimum value registered during the night. The daily characteristic RSAM values (in m s^−1^) are used by the authors to obtain the reduced average seismogram of the tremor time series. This reduced signal (known as RSAM or fumarolic tremor) includes the tremors generated by the entire fumarolic system at Pisciarelli, the subterraneous feeding channels and the surface vents. During the period of observation, the amplitude of fumarolic tremor at Pisciarelli increased by more than one order of magnitude with many peaks that, according to the authors, are linked to the hydrothermal fumarolic activity and reflects the increasing temperature/pressure conditions of the hydrothermal system.

Other experimental studies carried out at Stromboli (Italy)^17,18^, where continuous (strombolian) volcanic activity is present, measured gas velocity at fumaroles outlet of 10 m/s and pressure and thermal oscillations with frequency of ~2 Hz and average amplitude of 20 Pa and 30 °C.

In this paper we propose that a possible source of volcanic tremors is the oscillations of vapor bubbles confined within cavities of the hydrothermal basin surrounding the volcano. The idea is not new (see^1^) and can explain phenomena occurring at shallow depths (i.e. not exceeding few km from the ground surface), so that steam can separate from the liquid. The rocks hosting an hydrothermal system show a large amount of fractures of millimeters and sub-millimeters width and length from few centimeters up to meters (e.g.^19^) in which vapor bubbles can form. Bubbles spontaneous oscillations induced by heat flux have been a research topic in the last two centuries (see^20^). In the hydrothermal systems the heat source could be the hot fluid upraising in a fumarolic duct (e.g. Pisciarelli), or the magma that seeps in cavity, fractures and volcanic conduits. The magma, while equilibrating its temperature with the one of the host rocks, releases the heat needed to support the bubbles oscillations, that efficiently convert the thermal energy into mechanical energy. Since volcanic tremors of frequency 2–10 Hz are a very common phenomenon observed worldwide, they can be related to fundamental physical effects such as bubble oscillations. The general idea is that vapor bubbles develop near the heat source and, under proper conditions, dissipate energy not only by conduction but also by performing mechanical work (i.e. oscillating).

The dynamics of a single vapor bubble in an incompressible or nearly incompressible liquid is a famous problem. In the authors knowledge, the first theoretical model was introduced in^21^, who considered an incompressible fluid. The majority of these works is reviewed in^22^. We also acknowledge the study of^23^ devoted to the collapse of a spherical vapor bubble in a subcooled liquid, and the one of^24^ in wich the growth and collapse of a vapor bubble inside a microtube is experimentally and theoretically studied. Vapor bubbles dynamics in microchannels is considered also in^25^. The problem considers a heater element producing vapour and a cooler at the interface. The solution of this free boundary problem is found for a steady-state condition in a rectangular geometry. A number of studies have dealt with the growth or collapse of an axisymmetric bubble near a plane wall^26^ and with the instability problem of an oscillating spherical bubble^22^. Many important experimental and theoretical studies have investigated the problem of motion and deformation of cavitation bubbles in water^27–31^. The results obtained so far, show that the asymmetry produced for large bubbles (due to the shear stress close to the wall) affects the dynamic evolution of the bubble itself.

In this study we mimic the natural phenomenon using an experimental apparatus and then we develop a mathematical model capable of describing the observations. The experimental set-up reproduces in the lab the bubble oscillations occurring in a rock cavity. The experiment, described in Section 1, consists in a capillary tube filled with pure water and heated from the bottom. The lateral wall of tube is properly insulated, so that the heat flow occurs only from the bottom of the tube. When the water reaches the boiling temperature we observe the formation of a vapor bubble which grows up to a stable configuration around which it performs oscillations of small amplitude. We focus on this stage recording, by means of infrasonic detection system, the sound produced by the bubble oscillations. The analysis of the frequency spectrum revealed a peak in the spectrogram around 12 Hz.

To provide an insight on the bubble dynamics, a mathematical model of the system is presented in section 2. The aim of the model is to give a simple but rigorous description of the oscillatory behavior of this simple system that acts as a proxy for bubbles in fractures under a confining pressure (represented by the water column). The model is based on the energy balance between the liquid and vapor phase, where the vapor is treated as an ideal gas. To describe the phase transition between the two phases of the same single constituent (water) we have used the Clausius–Clapeyron relation. The FFT (Fast Fourier Transform) of the oscillating signal provided by the model exhibits a peak around 12 Hz, showing an excellent agreement with the spectrum obtained from the experimental measurements.

## The Experiment

The simple experiment proposed here, carried out in the IGG laboratories, evidences the dynamic of a vapor bubble on a lab scale. We designed this experiment to be as simple as possible using:

(i) An amorphous silica tube 1 m long, 8 mm diameter, wall thickness 1 mm. The amorphous silica tube is needed for safety, because it is able to resist to both the thermal stress and the shock induced by bubble vibrations;
(ii) Pure water (MilliQ grade);
(iii) Pyro-Bloc modules (Lynn Manufacturing inc.);
(iv) Bunsen burner (air-methane).
(v) Nikon D3000 buit-in condenser microphone.
(vi) OriginLab software for the determination of the spectrogram of the recorded signal.

The diameter of the silica tube is not very important as long as the tube diameter is small enough to prevent convective motion but sufficiently large to minimize the meniscus effect. During our experiments, we tested different pipes (6, 8, 10, 12 mm of diameter) and in the 12 mm tube diameter the phenomenon do not present, likely due the bubbles upraising and generating convective cells.

**Figure 1:**
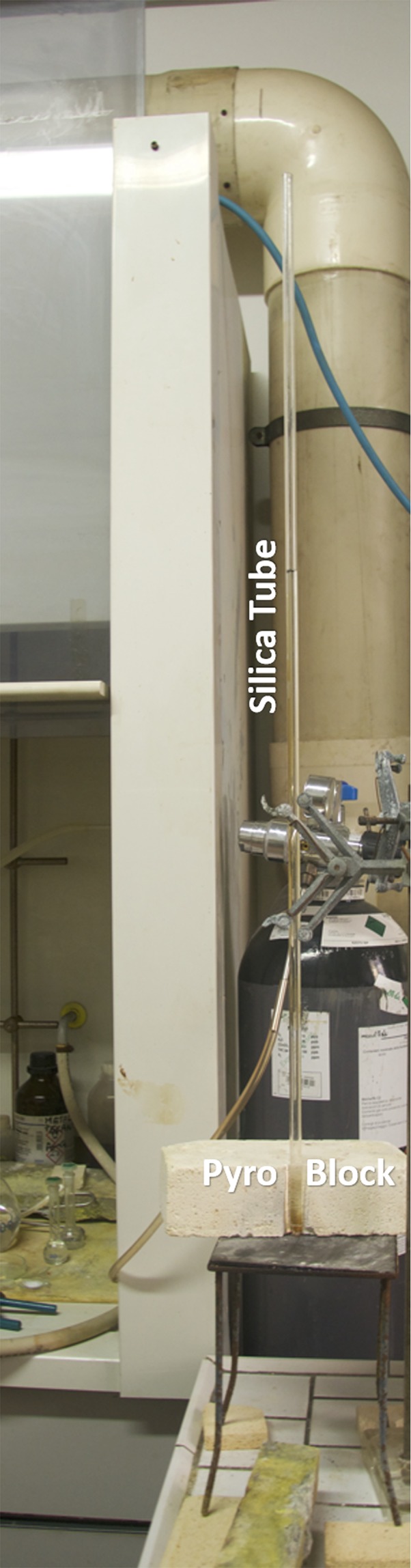
Experimetal apparatus. Silica tube is 75 cm long, 8 mm diameter, 1 mm thickness. Pyro Block height is 5 cm, size 20 × 20 cm. Bunsen Burner have a methane/air oxydizing flame.

**Figure 2:**
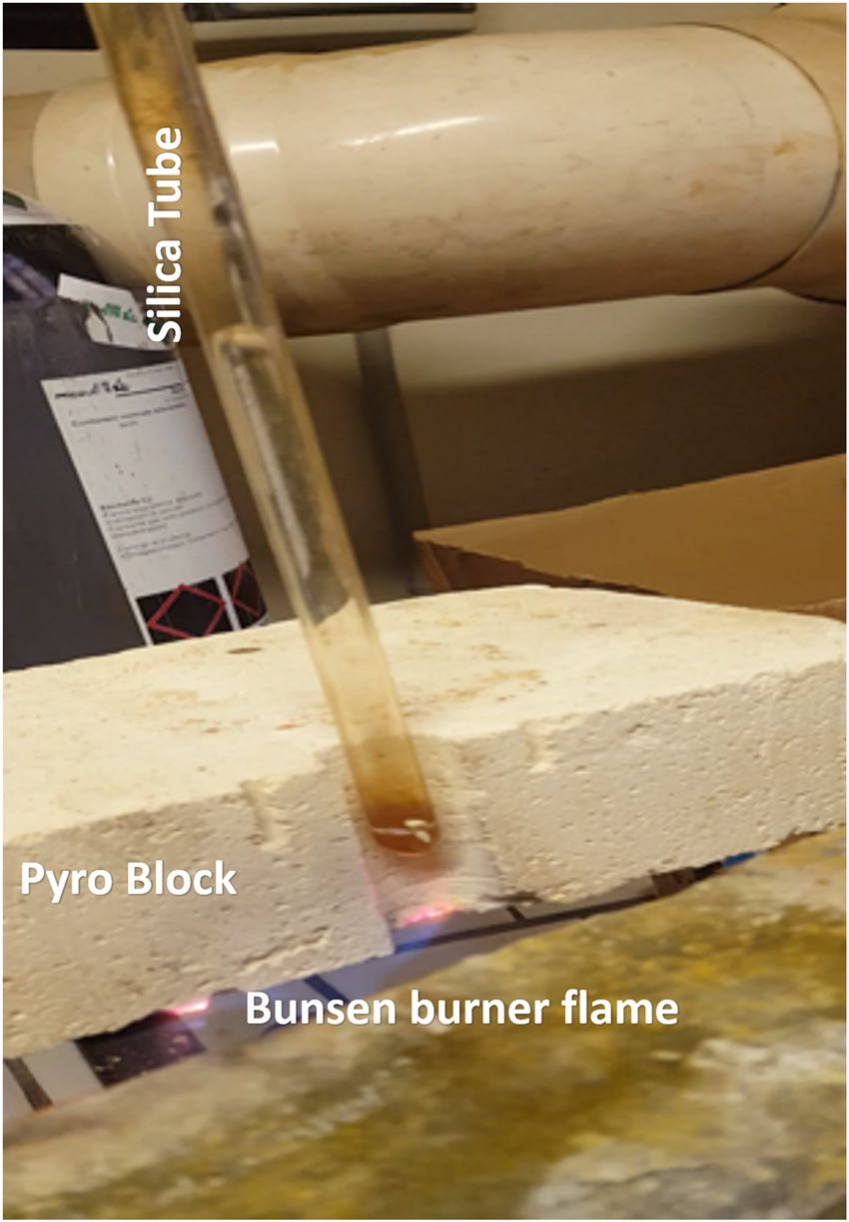
Close up, with the bunsen burner flame heating from below. The pyro block is cut in two to show the hole hosting the silica tube.

The tube is suspended over a Bunsen burner and then inserted in a 5 cm thick PyroBloc module where a hole, having the same diameter of the tube, has been previously drilled. The tube is heated from the bottom since the 5 cm thickness PyroBloc practically avoids any lateral heating. The silica tube is filled with pure water (MilliQ grade) up to nearly 60 cm. After having set up the system, the Bunsen burner is turned on and positioned with the oxidizing flame at the tube bottom (Figs 1 and 2).

After approximately 30 minutes a vapor bubble of nearly 3 cm height is formed at the bottom of the tube. For about 30 minutes after the bubble forming, the system performs small oscillations. The vapor bubble acts as a “spring” for the liquid column above it, which performs oscillations with amplitude of the order of a few millimeters. Then the bubble collapses and, in a relatively short time, a second bubble which grows very rapidly is formed. The expansion of the second bubble eventually produces a sort of “explosion” that makes the water column outpour from the tube.

**Figure 3:**
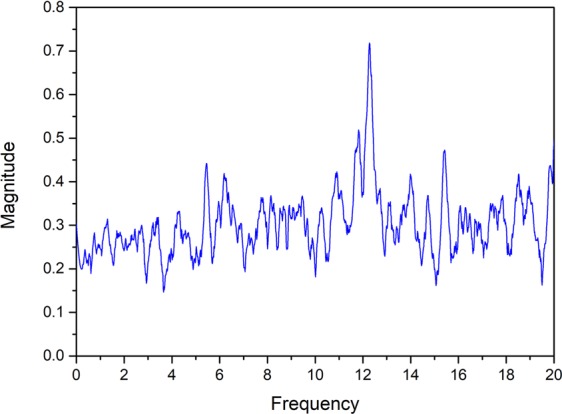
Spectrogram of a 5 minutes period of the acoustic signal recorded during the experiment.

The experimental data shown here refer to the second stage, that we named “oscillatory stage”. In Fig. 3 the spectrogram of the recorded periodic signal is reported, showing a peak around 12 Hz.

## The Mathematical Model

**Figure 4:**
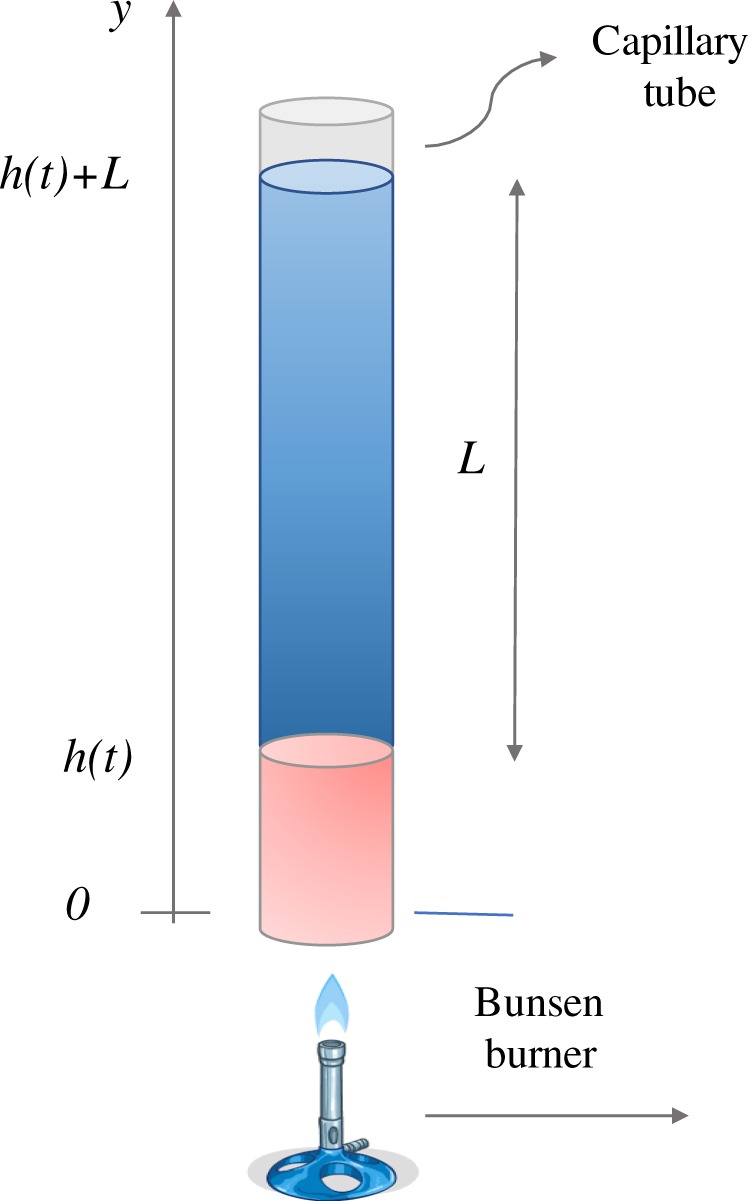
Schematic representation of the system. In blue is evidenced the liquid column, in pink the vapour phase after the bubble has formed. The bunsen burner provides the heat flow from the bottom.

We consider a one dimensional model as the one depicted in Fig. 4, and focus on the second stage (i.e. the “oscillatory stage”), so that the initial time *t* = 0, corresponds to the time at which the vapor bubble has just formed. We denote by *L* the height of the liquid column and by *y* the vertical coordinate (pointing upward) so that *y* = 0 is the tube bottom and *y* = *h*(*t*) represents the height of the vapor bubble. We assume that in the vapor phase, [0, *h*], the physical variables do not depend on *y* and model the liquid column, [*h*, *h* + *L*], as a body moving with uniform velocity. The mathematical problem is formulated writing the energy balance for the liquid and vapor phase (coupled with appropriate interface conditions) and the motion equation for liquid column. We remark that on the interface *h*(*t*) (which is a free boundary) a vapor-liquid phase transitions takes place, so that *h*(*t*) is not a material interface.

### **The liquid phase**

Neglecting convective motions the energy equation in the liquid column yields

1 $${\rho }_{l}{c}_{l}(\frac{\partial {\theta }_{l}}{\partial t}+\dot{h}(t)\frac{\partial {\theta }_{l}}{\partial y})={k}_{l}\frac{{\partial }^{2}{\theta }_{l}}{\partial {y}^{2}},$$

where: *θ*_*l*_(*y*, *t*) is the water temperature, $\dot{h}(t)$ is the vapor-liquid interface vertical velocity and *ρ*_*l*_, *c*_*l*_, *k*_*l*_ are the density, specific heat, heat conductivity of water. The boundary and initial conditions are

2 $${\theta }_{l}(h+L,t)={\theta }_{a},\,{\theta }_{l}(h,t)=\theta (t),\,{\theta }_{l}(y,0)={\theta }_{in}(y),$$

where *θ*_*a*_ is the ambient temperature, *θ*(*t*) is the temperature of the vapor in the bubble and *θ*_*in*_(*y*) is the temperature profile at *t* = 0. We remark that (2)_2_ provides the coupling between the temperature field in the water and the the bubble vapor temperature whose evolution equation will be illustrated in Section 2.2. Introducing the transformation

$$\xi =y-h(t),$$

and the new variable

$$\Theta (\xi ,t)={\theta }_{l}(\xi +h,t),$$

Equation (1) and conditions (2) become

3 $$\frac{\partial \Theta }{\partial t}=D\frac{{\partial }^{2}\Theta }{\partial {\xi }^{2}},\,0\le \xi \le L,$$

4 $$\Theta (L,t)={\theta }_{a},\,\Theta (0,t)=\theta (t),\,\Theta (\xi ,0)={\theta }_{in}(\xi +{h}_{o}),$$

where

5 $$D=\frac{{k}_{l}}{{\rho }_{l}{c}_{l}},$$

is the water thermal diffusivity and where *h*_*o*_ is the “equilibrium” height of the vapor bubble, i.e. the height around which the oscillations occur. The quantity *h*_*o*_ will be also used as the reference height of the vapor bubble.

### **The vapor bubble**

We model the vapor as an ideal gas in which temperature and pressure are linked by Clayperon’s equation^32^. Hence we write

6 $$\{\begin{array}{rcl}p & = & {p}_{o}\exp [-\frac{\lambda }{r}(\frac{1}{\theta }-\frac{1}{{\theta }_{o}})],\\ p & = & r\rho \theta ,\end{array}$$

where *p* is the vapor pressure, *ρ* is the vapor density, *λ* is the latent heat of vaporization, *r* is the specific gas constant and *θ*_*o*_ is the evaporation temperature at the reference pressure *p*_*o*_. From (6) we see that

7 $$\rho (\theta )=\frac{{p}_{o}}{r\theta }\exp [-\frac{\lambda }{r}(\frac{1}{\theta }-\frac{1}{{\theta }_{o}})],$$

and

8 $$\frac{dp}{d\theta }=\frac{p\lambda }{r{\theta }^{2}}=\frac{\rho \lambda }{\theta }\mathrm{.}$$

### **Energy balance**

From the first principle of thermodynamics the change in internal energy of the vapor is given by

9 $$dU=\delta Q-p\,dV,$$

where *δQ* is the infinitesimal increment of heat supplied to the system and *pdV* is the infinitesimal work done by the system, *V* being the volume of the bubble. We notice that *V*(*t*) = *Ah*(*t*), where *A* is the cross section area of the capillary tube. In our case, since the lateral wall is adiabatic and no heat source/sink is present, we have

$$\delta Q=({\dot{Q}}_{b}-{\dot{Q}}_{h})dt,$$

where ${\dot{Q}}_{b}$ > 0 is the heat supplied at the bottom surface by the bunsen burner (constant in time) and ${\dot{Q}}_{h}$ is the heat flux through the interface *y* = *h*(*t*). The infini*t*esimal variation of internal energy *dU* in the vapor phase is given by *dU* = *d*(*ρVc*_*v*_*θ*), where *c*_*v*_ is the spe*c*ific heat at constant volume. Recalling that *c*_*p*_ − *c*_*v*_ = *r* (*c*_*p*_ being the s_*p*_ecific heat at constant pressure) (9) rewrites as

10 $$d[\rho V({c}_{p}-r)\theta ]=({\dot{Q}}_{b}-{\dot{Q}}_{h})dt-pdV.$$

Exploiting (6) and (8) we write

$$pdV=d(pV)-dpV=d(r\rho \theta V)-\frac{\lambda \rho V}{\theta }d\theta ,$$

so that (10) becomes

11 $$\rho V({c}_{p}-\frac{\lambda }{\theta })d\theta +{c}_{p}\theta d(\rho V)=({\dot{Q}}_{b}-{\dot{Q}}_{h})dt,$$

where *ρV* is the mass of the vapor within the bubble. To close Eq. (11) we need an expression for ${\dot{Q}}_{h}$. To this aim, we recall that *h*(*t*) is an evaporation/condensation interface: here the heat fluxes have a jump proportional to the heat absorbed, or released, by the phase change. Applying the Stefan condition (see^33^ or^34^)

$${\dot{Q}}_{h}dt=\lambda d(\rho V)+{\dot{Q}}_{l}dt,$$

where ${\dot{Q}}_{l}$ is the heat flux of the liquid phase at the interface *y* = *h* which, exploiting Fourier’s law, is given by

$${\dot{Q}}_{l}=-\,{k}_{l}A{\frac{\partial {\theta }_{l}}{\partial y}|}_{y=h},$$

with *θ*_*l*_ obtained solving (3), (4). In conclusion Eq. (11) can be rewritten in the following form

12 $$\rho V({c}_{p}-\frac{\lambda }{\theta })\frac{d\theta }{dt}+({c}_{p}\theta +\lambda )\frac{d(\rho V)}{dt}={\dot{Q}}_{b}+{k}_{l}A{\frac{\partial {\theta }_{l}}{\partial y}|}_{y=h},$$

or equivalently

13 $$\frac{\rho Ah\lambda }{\theta }(\frac{{c}_{p}\theta }{\lambda }-1)\frac{d\theta }{dt}+\lambda A(\frac{{c}_{p}\theta }{\lambda }+1)\frac{d(\rho h)}{dt}={\dot{Q}}_{b}+{k}_{l}A{\frac{\partial \Theta }{\partial \xi }|}_{\xi =0}.$$

### **Momentum balance for the liquid column**

The momentum equation for the liquid column is given by

14 $${m}_{l}\frac{{d}^{2}h}{d{t}^{2}}=-\,{m}_{l}g-{p}_{a}A+pA-\beta \frac{dh}{dt},$$

where

15 $${m}_{l}={\rho }_{l}AL,$$

is the mass of the liquid column, *p*_*a*_ is the atmospheric pressure acting on the free surface of the liquid column and where the last term represents the damping due to viscous friction. The right hand side of (14) is therefore the net force acting on the water column. Choosing the reference pressure as (the reference pressure is thus the sum of the ambient pressure and the pressure exerted by the water column)

16 $${p}_{o}=\frac{{m}_{l}g}{A}+{p}_{a}={\rho }_{l}g(L+H),$$

with *H* ≈ 10 m, and recalling (6), we may rewrite (14) as

17 $${m}_{l}\frac{{d}^{2}h}{d{t}^{2}}={p}_{o}A\{\exp [-\frac{\lambda }{r}(\frac{1}{\theta }-\frac{1}{{\theta }_{o}})]-1\}-\beta \frac{dh}{dt}.$$

### **The non dimensional problem**

Let us rescale the problem with non dimensional variables. We introduce

$$\xi =L\tilde{\xi },\,h={h}_{o}\tilde{h},\,V=A{h}_{o}\tilde{h},\,t={t}_{c}\tilde{t},$$

where *t*_*c*_ is a characteristic time still to be selected and *h*_*o*_ is the characteristic height of the bubble. Next we set

$$\theta ={\theta }_{o}\tilde{\theta },\,\Theta ={\theta }_{o}\tilde{\Theta },\,p={p}_{o}\tilde{p},\,\rho ={\rho }_{o}\tilde{\rho },$$

with *p*_*o*_ reference pressure given by (16), *θ*_*o*_ the water boiling temperature at pressure *p*_*o*_ and

18 $${\rho }_{o}=\frac{{p}_{o}}{r{\theta }_{o}},$$

reference vapor density. In particular, (7) rewrites as

19 $$\tilde{\rho }=\frac{1}{\tilde{\theta }}\exp \{-\frac{\lambda }{r{\theta }_{o}}(\frac{1}{\tilde{\theta }}-1)\}.$$

Equation (3) becomes

$$\frac{\partial \tilde{\Theta }}{\partial \tilde{t}}=(\frac{{t}_{c}}{{t}_{D}})\frac{{\partial }^{2}\tilde{\Theta }}{\partial {\tilde{\xi }}^{2}},$$

where *t*_*D*_ = *L*^2^*D*^−1^ is the characteristic diffusive time in the water. The boundary and initial conditions become

$${\tilde{\Theta }|}_{\tilde{\xi }=1}=\frac{{\theta }_{a}}{{\theta }_{o}}={\tilde{\Theta }}_{a},\,\,{\tilde{\Theta }|}_{\tilde{\xi }=0}=\tilde{\theta },\,\,\tilde{\Theta }(\tilde{\xi },0)=\frac{{\theta }_{in}(\tilde{\xi }L+{h}_{o})}{{\theta }_{o}}={\tilde{\Theta }}_{in}(\tilde{\xi }).$$

We then we introduce

$${t}_{b}=(\frac{{\rho }_{o}A{h}_{o}\lambda }{{\dot{Q}}_{b}}),\,{t}_{h}=(\frac{{\rho }_{o}{h}_{o}L\lambda }{{k}_{l}{\theta }_{o}}),$$

where *t*_*b*_ is the characteristic time of the heat supply due to the bunsen burner and *t*_*h*_ is the characteristic time of phase transition, so that (13) becomes

20 $$\frac{\tilde{\rho }\tilde{h}}{\tilde{\theta }}(\frac{{c}_{p}{\theta }_{o}}{\lambda }\tilde{\theta }-1)\frac{d\tilde{\theta }}{d\tilde{t}}+(\frac{{c}_{p}{\theta }_{o}}{\lambda }\tilde{\theta }+1)\frac{d(\tilde{\rho }\tilde{h})}{d\tilde{t}}=(\frac{{t}_{c}}{{t}_{b}})+(\frac{{t}_{c}}{{t}_{h}}){\frac{\partial \tilde{\Theta }}{\partial \tilde{\xi }}|}_{\tilde{\xi }=0}\mathrm{.}$$

Next, we introduce the new dependent variable

$$\tilde{\zeta }(\tilde{t})=\frac{{\tilde{\zeta }}_{o}}{\tilde{\theta }(\tilde{t})},\,{\rm{with}}\,{\tilde{\zeta }}_{o}=\frac{\lambda }{r{\theta }_{o}},$$

and the heat capacity ratio

$$\gamma =\frac{{c}_{p}}{{c}_{v}},\,\frac{r}{{c}_{p}}=\frac{\gamma -1}{\gamma },$$

so that (20) rewrites as

21 $$\tilde{\rho }\tilde{h}(1-\frac{1}{\tilde{\zeta }}\frac{\gamma }{\gamma -1})\frac{1}{\tilde{\zeta }}\frac{d\tilde{\zeta }}{d\tilde{t}}+(1+\frac{1}{\tilde{\zeta }}\frac{\gamma }{\gamma -1})\frac{d(\tilde{\rho }\tilde{h})}{d\tilde{t}}=(\frac{{t}_{c}}{{t}_{b}})+{(\frac{{t}_{c}}{{t}_{h}})\frac{\partial \tilde{\Theta }}{\partial \tilde{\xi }}|}_{\tilde{\xi }=0},$$

where

22 $$\tilde{\rho }=\frac{\tilde{\zeta }}{{\tilde{\zeta }}_{o}}\exp ({\tilde{\zeta }}_{o}-\tilde{\zeta }\mathrm{).}$$

Note that

$$\frac{d(\tilde{\rho }\tilde{h})}{d\tilde{t}}=\frac{d\tilde{\rho }}{d\tilde{t}}\tilde{h}+\frac{d\tilde{h}}{d\tilde{t}}\tilde{\rho }=\tilde{\rho }\tilde{h}\frac{d\tilde{\zeta }}{d\tilde{t}}(\frac{1}{\tilde{\zeta }}-1)+\frac{d\tilde{h}}{d\tilde{t}}\tilde{\rho },$$

so that (21) can be rewritten as

$$\tilde{\rho }\tilde{h}(2-\tilde{\zeta }-\frac{\gamma }{\gamma -1})\frac{1}{\tilde{\zeta }}\frac{d\tilde{\zeta }}{d\tilde{t}}+(1+\frac{1}{\tilde{\zeta }}\frac{\gamma }{\gamma -1})\tilde{\rho }\frac{d\tilde{h}}{d\tilde{t}}=(\frac{{t}_{c}}{{t}_{b}})+(\frac{{t}_{c}}{{t}_{h}})\frac{\partial \tilde{\Theta }}{\partial \tilde{\xi }}\mathrm{(0,}\,t\mathrm{).}$$

To write the non dimensional version of the momentum balance of the water column we introduce the characteristic oscillation time

23 $${t}_{o}=\sqrt{\frac{{m}_{l}{h}_{o}}{{m}_{l}g+{p}_{a}A}}=\sqrt{\frac{{m}_{l}{h}_{o}}{A{p}_{o}}}=\sqrt{\frac{{h}_{o}}{g(1+H{L}^{-1})}},$$

and the characteristic damping time

$${t}_{d}=(\frac{{m}_{l}}{\beta }),$$

so that Eq. (17) reduces to

24 $$\frac{{d}^{2}\tilde{h}}{d{\tilde{t}}^{2}}={(\frac{{t}_{c}}{{t}_{o}})}^{2}[\exp ({\tilde{\zeta }}_{o}-\tilde{\zeta })-1]-(\frac{{t}_{c}}{{t}_{d}})\frac{d\tilde{h}}{d\tilde{t}}\mathrm{.}$$

Since we are interested in the oscillation of the bubble we select *t*_*o*_ as characteristic time, that is *t*_*c*_ = *t*_*o*_. We end up with the following mathematical problem for the unknowns (We omit the “~” to keep notation light) Θ(*ξ*, *t*), *ζ*(*t*) and *h*(*t*)

25 $$\{\begin{array}{l}\frac{\rho h}{\zeta }(2-\zeta -\frac{1}{\zeta }\frac{\gamma }{\gamma -1})\frac{d\zeta }{dt}+(1+\frac{1}{\zeta }\frac{\gamma }{\gamma -1})\rho \frac{dh}{dt}=\alpha +\delta {\frac{\partial \Theta }{\partial \xi }|}_{\xi =0},\\ \frac{{d}^{2}h}{d{t}^{2}}=[\exp ({\zeta }_{o}-\zeta )-1]-\chi \frac{dh}{dt},\\ \frac{\partial \Theta }{\partial t}=\nu \frac{{\partial }^{2}\Theta }{\partial {\xi }^{2}},\end{array}$$

where the values of

$$\alpha =(\frac{{t}_{o}}{{t}_{b}}),\,\delta =(\frac{{t}_{o}}{{t}_{h}}),\,\chi =(\frac{{t}_{o}}{{t}_{d}}),\,\nu =(\frac{{t}_{o}}{{t}_{D}}),$$

reported in Table 2 are determined using the physical parameters of Table 1. The initial and boundary conditions are

26 $$\{\begin{array}{l}\Theta (\xi ,0)={\Theta }_{in}(\xi ),\,\zeta (0)={\zeta }_{in},\,h(0)=1,\\ \Theta (0,t)=\frac{{\zeta }_{o}}{\zeta },\,\Theta (1,t)={\Theta }_{a}.\end{array}$$

**Table 1:** Dimensional parameters.

Parameter	Value	Dimensions	Description
*ρ*_*o*_	0.59776	Kg/m^3^	vapor density at *p*_*o*_ and *θ*_*o*_
*ρ*_*l*_	0.9982071	Kg/m^3^	density of the water
*p*_*a*_	101325	Kg/(m⋅s^2^)	atmospheric pressure
*A*	5.0265 · 10^−5^	m^2^	cross section area of the tube
*h*_*o*_	3 · 10^−2^	m	initial height of the vapor bubble
*g*	9.80665	m/s^2^	gravity constant
${\dot{Q}}_{b}$	1 · 10^−3^	W	bunsen heat flux
*L*	6 · 10^−1^	m	height of the water column
*λ*	2.272 · 10^6^	J/Kg	water latent heat of vaporization
*k*_*l*_	0.6	W/(m⋅°K)	water heat conductivity
*c*_*l*_	4.186 · 10^3^	J/(Kg⋅°K)	water specific heat
*θ*_*o*_	372.2	°K	vapor temperature at *p*_*o*_
*θ*_*a*_	293.16	°K	ambient temperature
*μ*	1.0016 · 10^−3^	Kg/(m⋅s)	water viscosity
*r*	4.616 · 10^2^	J/(Kg⋅°K)	vapor specific gas constant
*c*_*p*_	1.94 · 10^3^	J/(Kg⋅°K)	vapor specific gas constant
*c*_*v*_	1.4784 · 10^3^	J/(Kg⋅°K)	vapor specific gas constant
*H*	10	m	water column height corresponding to atmospheric pressure

System (25) is quite involved because two nonlinear Ordinary Differential Equations (ODEs) are coupled with a parabolic equation for Θ. We however remark that $\nu  \sim {10}^{-9}$, and so only a thin layer of water facing the interface is heated by the vapor. This allows to approximate the heat flux by (see Appendix A)

27 $${\frac{\partial \Theta }{\partial \xi }|}_{\xi =0}=\mathop{\underbrace{-\frac{1}{\sqrt{\pi \nu t}}[(\frac{{\zeta }_{o}}{\zeta (t)}-1)+(1-{\Theta }_{a})\exp (-\frac{{\xi }_{d}^{2}}{4\nu t})],}}\limits_{F(\zeta ,t)}$$

where *ξ*_*d*_ is the rescaled thickness of the thermal boundary layer in which temperature decreases from the boiling temperature to ambient temperature. Setting *v* = $\dot{h}$, the system (25) can be simplified getting to the following first-order system of ODE’s

28 $$\{\begin{array}{rcl}\dot{h} & = & v,\\ \dot{v} & = & \exp ({\zeta }_{o}-\zeta )-1-\chi v,\\ \dot{\zeta } & = & \zeta \frac{\alpha +\delta F(\zeta ,t)-(1+\frac{1}{\zeta }\frac{\gamma }{\gamma -1})\rho (\zeta )z}{\rho (\zeta )h(2-\zeta -\frac{\gamma }{\gamma -1})},\end{array}$$

where *ρ*(*ζ*) is given by (22) and *F*(*ζ*, *t*) is given by (27).

## Numerical Results

Here we plot the solution of system (28) using the parameters of Tables 1 and 2 and using the following initial data

$$h(0)=1\,v(0)=1e-04\,\zeta (0)={\zeta }_{o}=13.2241$$

**Table 2:** Characteristic times and non-dimensional parameters.

characteristic time	value	parameter	value
*t*_*o*_	0.0138 s	*α*	5.9438 · 10^−6^
*t*_*b*_	2.3208 · 10^3^ s	*δ*	1.0158 · 10^−8^
*t*_*h*_	1.3580 · 10^6^ s	*χ*	1.3816 · 10^−4^
*t*_*d*_	99.8393 s	*ν*	5.4922 · 10^−9^
*t*_*D*_	2.5116 · 10^6^ s	Θ_*a*_	7.81 · 10^−1^
		*γ*	1.3122
		*ζ*_*o*_	13.2241

**Figure 5:**
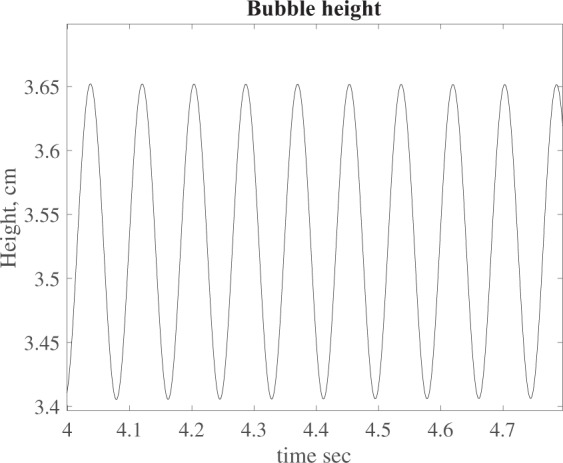
Bubble height vs. time.

**Figure 6:**
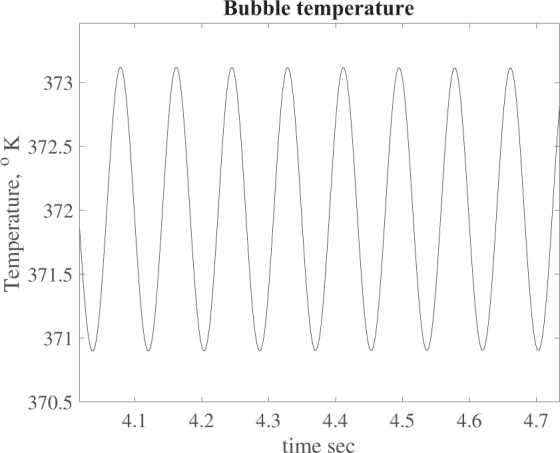
Bubble temperature vs time.

**Figure 7:**
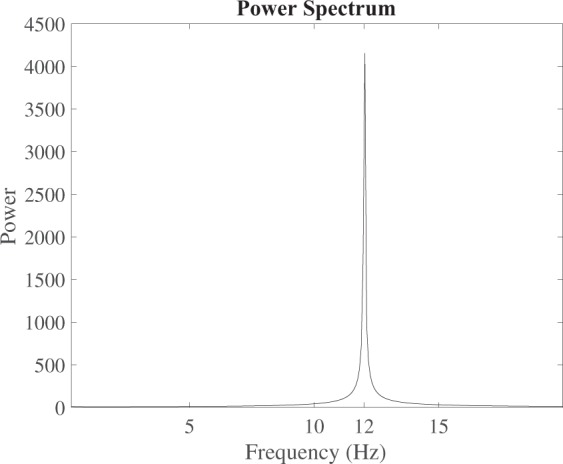
Computed power spectrum.

In particular we plot the height *h*(*t*) and the temperature *θ*(*t*) of the bubble as a function of time, see Figs 5 and 6. Finally we perform a FFT (Fast Fourier Transform) of the oscillation signals and evaluate the frequency domain. The power spectrum is shown in Fig. 7 where we can see a peak at ~12 Hz, i.e. at the same frequency recorded during the experiment (see Fig. 3). In particular, Fig. 6 shows that the bubble temperature is essentially constant during the oscillations, meaning that the phase change process has little influence on the dynamics. So, we can consider

29 $${\rho }_{o}{h}_{o}=\rho h,$$

with *ρ*_*o*_ given by (18) and

30 $$\frac{{p}_{o}}{{\rho }_{o}}=\frac{p}{\rho },$$

*p*_*o*_ being the “equilibrium” pressure given by (16). This fact allows to simplify considerably the mechanical model (14). Indeed, neglecting the damping term, Eqs (14), (6)_2_, (29) and (30) yield

$${m}_{l}\frac{{d}^{2}h}{d{t}^{2}}=\mathop{\underbrace{-{m}_{l}g-{p}_{a}A}}\limits_{-{p}_{o}A}+\mathop{\underbrace{pA}}\limits_{A{p}_{o}\frac{\rho }{{\rho }_{o}}}=A{p}_{o}(\frac{{h}_{o}}{h}-1)\mathrm{.}$$

Recalling (15), (23) and setting *h*(*t*) = *h*_*o*_(1 + *y*(*t*)), we have

31 $$1+\ddot{y}=\frac{1}{{t}_{o}^{2}}(\frac{1}{1+y}-1).$$

Assuming that $y\ll 1$ we approximate the above with

$$\ddot{y}+\frac{y}{{t}_{o}^{2}}=-\,1.$$

whose frequency of oscillation is

32 $$\nu =\frac{1}{2\pi {t}_{o}}=\frac{1}{2\pi }\sqrt{\frac{g}{{h}_{o}}(1+\frac{H}{L})}.$$

Referring to the data of Table 1 we obtain *ν* ≈ 12 Hz, i.e. the recorded frequency and the output of the complete model (28). In particular, (32) leads to some of observations that we shall discuss in the next section.

## Discussion and Conclusions

The formation of bubbles in a liquid is an efficient way of mechanical energy generation and this fact has been recognized in geophysics^35^. Boiling of groundwater causes the formation and growth of bubbles in the liquid permeating the rocks of the geothermal and volcanic system. The bubbles start to oscillate if supported by a suitable heat flux. In particular, the bubbles oscillation occurs at a frequency which is derived neglecting both the phase change process and the temperature variations. So, from (14) we derive (32). Equation (31) can be related to Mathieu equation^36^ that is known to produce resonance. In our interpretation, among the many possible oscillating frequencies occurring in the natural system, it is likely that a number of different bubbles begin to resonate producing a harmonic tremor at a given frequency.

**Figure 8:**
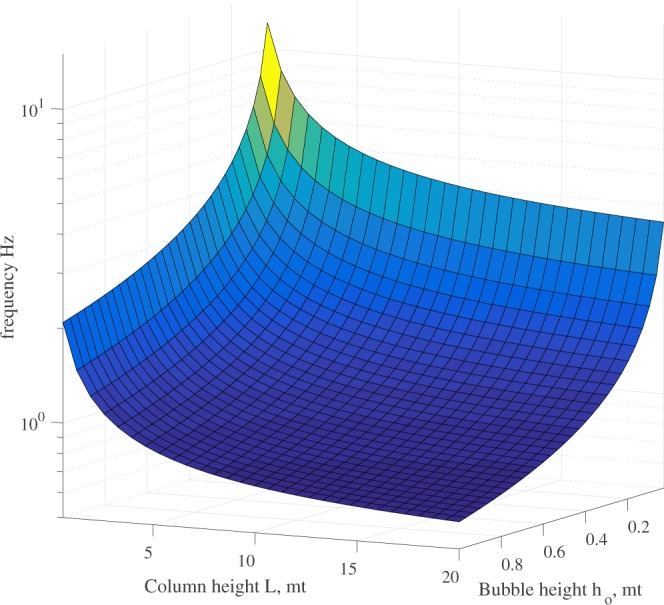
Plot of the function *ν* given by (32) as a function of *h*_*o*_ and *L*.

As one can easily recognize from (32), the frequency is a function of the characteristic bubble height and the column length. A 3D plot of the function (32) is shown in Fig. 8. The pair (*h*_*o*_, *L*) spans in the domain [0.01, 1] × [0.5, 20] meaning that we are considering 1 cm as the minimum height of the bubble and 1 *m* as the maximum, while minimum *L* is 0.5 m and maximum *L* is 20 m. As one can see, the frequency range is [0.6, 12.09] which is exactly the range of the frequencies for harmonic tremors recorded all over the world. To provide a better understanding on how we may accurately model the measured frequency for bubble oscillation and the effect of *H*/*L* in Eq. (32), we model the fumarolic tremor measured by^16^ at Pisciarelli fumarole, where the frequency at fumarole head is nearly 10 Hz. We model the system as our lab experiments, using the same conditions (the real conditions are very similar, only the temperature is higher) with a *H*/*L* ratio of 0.056667. Setting *Q*_*h*_ = 0.1 W (see Table 1), we are able to reproduce the measured frequency span.

The heat flux affects the timespan of the phenomenon, meaning that *Q*_*h*_ = 10^−3^ W (see Table 1) is sufficient to sustain the oscillation, while with larger values of *Q*_*h*_ the bubble size increases, modifying the oscillation frequency, until it disappears. This effect provides a frequency span for the oscillation of a single bubble, that in the natural system could overlap with the resonance between different bubbles oscillating at different frequencies. At Vulcano, Vulcano Island (Italy)^37^ many seismic signals related to bubbles in the hydrothermal system are detected. In particular, these signals are produced by fluid flow inside cracks and conduits with frequencies that depend on the gas volume fraction. Among them there are monochromatic events that show a single frequency peak at 6 or 8 Hz and generally last about 10–15 s, with few of them lasting up to 30–50 s with a slow amplitude decay. According to the author, these features are highly similar to the so-called “tornillos” found in other volcanoes (Galeras^38^, Tongariro^39^, Tatun Volcano Group^40^).

To model the 6–8 Hz oscillations we need a *H*/*L* (parameters from (32) and Table 1) ratio of 0.05, with a heat flux *Q*_*h*_ of 0.1 W. If we use a *H*/*L* of 0.0125 with an heat flux *Q*_*h*_ of 0.1 (more than 50 s) or 0.4 to reduce the lifetime of the phenomenon, we obtain oscillations in a frequency range of 2–4 Hz corresponding to the higher infrasonic signal frequency detected in^37^. Ripepe *et al*.^4^ measured infrasonic waves with similar spectral content between 2 and 10 Hz at Stromboli volcano crater. The peaks centered at 5 Hz can be attributed to gas bubbles expansion, that is the phenomenon studied in this paper. Therefore we may state that our model is capable of reproducing the measured infrasound signals for the case study of Solfatara (Pisciarelli fumarole), Vulcano (Vulcano Island) and Stromboli. In particular, the case of Vulcano covers typical infrasound signals found in many other volcanoes around the world. In this work, we are able to reproduce the measured infrasound signals from different volcanoes (namely Solfatara at Pisciarelli fumarole, Vulcano, Stromboli) in which the infrasound signals are attributed to bubble oscillations in cracks or in magma (Stromboli). We remind that one of the main challenge is to prove the relation between the emitted infrasound and the bubble oscillations, but once this is done we are able to accurately reproduce the signal frequency with our model.

## Appendix A

Here we show a procedure to get an approximated expression for Θ_*ξ*_(0, *t*) appearing in the system (25). We assume that the initial thermal profile in the liquid can be approximated by

33 $$\Theta (\xi ,0)=\{\begin{array}{cc}1 & 0\le {\xi }_{d} < \xi ,\\ {\Theta }_{a} & {\xi }_{d}\le \xi \le 1,\end{array}$$

where we recall that Θ_*a*_ is the rescaled ambient temperature. The above means that in a layer of thickness *ξ*_*d*_ the temperature of the liquid is the boiling temperature, while outside the layer the fluid is everywhere at ambient temperature. A reasonable value for *ξ*_*d*_ is 1/60. Indeed, recalling that the length of the column is 60 cm, the assumption *ξ*_*d*_ = 1/60 means that we have 1 cm of the water column close to the bubble at the boiling temperature, while the rest is essentially at ambient temperature. Introducing the new non dimensional variables

$$\eta =\frac{\xi }{\sqrt{\nu }}\,v(\eta ,t)=\Theta (\xi ,t)-{\Theta }_{a},$$

we get (see Table 2)

$$\xi =1\,\Rightarrow \,\eta ={\nu }^{-1/2}=O({10}^{4})\gg 1.$$

As a consequence the problem for *v*(*η*, *t*) becomes

34 $$\{\begin{array}{rr}\begin{array}{c}\frac{\partial v}{\partial t}=\nu \frac{{\partial }^{2}v}{\partial {\eta }^{2}}\\ v(\eta ,\,\mathrm{0)}={v}_{in}(\eta )\\ v\mathrm{(0,}\,t)=\frac{{\zeta }_{o}}{\zeta }-{\varTheta }_{a}\\ v(\infty ,t)=0\end{array} & \begin{array}{c}\eta  > \mathrm{0,}\,\,t > \mathrm{0,}\\ \eta  > \mathrm{0,}\\ t > \mathrm{0,}\\ t > 0.\end{array}\end{array}$$

where

35 $${v}_{in}(\eta )=\{\begin{array}{cc}1-{\Theta }_{a} & 0\le {\eta }_{d} < \eta ,\\ 0 & {\eta }_{d}\le \eta ,\end{array}$$

and where

$${\eta }_{d}=\frac{1}{60\sqrt{\nu }}\approx 190$$

Following^41^ we can easily check that the explicit solution to (34) is given by

36 $$\begin{array}{c}v(\eta ,t)=(\frac{{\zeta }_{o}}{\zeta (t)}-{\Theta }_{a})-(\frac{{\zeta }_{o}}{\zeta (t)}-1){\rm{erf}}(\frac{\eta }{2\sqrt{t}})\\ \,\,\,\,+-\frac{1-{\Theta }_{a}}{2}[{\rm{erf}}(\frac{\eta -{\eta }_{d}}{2\sqrt{t}})+{\rm{erf}}(\frac{\eta +{\eta }_{d}}{2\sqrt{t}})]\end{array}$$

Differentiating w.r.t. the space variable and going back to the original unknown Θ(*ξ*, *t*) one can easily check that

37 $$\frac{\partial \Theta }{\partial \xi }(0,t)=-\,\frac{1}{\sqrt{\pi \nu t}}[(\frac{{\zeta }_{o}}{\zeta (t)}-1)+(1-{\Theta }_{a})\exp (-\frac{{\xi }_{d}^{2}}{4\nu t})].$$
